# Unique patterns of transcript and miRNA expression in the South American strong voltage electric eel (*Electrophorus electricus*)

**DOI:** 10.1186/s12864-015-1288-8

**Published:** 2015-03-26

**Authors:** Lindsay L Traeger, Jeremy D Volkening, Howell Moffett, Jason R Gallant, Po-Hao Chen, Carl D Novina, George N Phillips, Rene Anand, Gregg B Wells, Matthew Pinch, Robert Güth, Graciela A Unguez, James S Albert, Harold Zakon, Michael R Sussman, Manoj P Samanta

**Affiliations:** Department of Genetics, University of Wisconsin, Madison, WI 53706 USA; Biotechnology Center, University of Wisconsin, Madison, WI 53706 USA; Department of Biochemistry, University of Wisconsin, Madison, WI 53706 USA; Department of Cancer Immunology and AIDS, Dana-Farber Cancer Institute, Boston, MA 02115 USA; Department of Microbiology and Immunobiology, Harvard Medical School, Boston, MA 02115 USA; Department of Zoology, Michigan State University, East Lansing, MI 48824 USA; BEACON Center for the Study of Evolution in Action, Lansing, USA; Broad Institute of Harvard and MIT, Cambridge, MA 02141 USA; BioSciences at Rice and Department of Chemistry, Rice University, Houston, TX 77005 USA; Department of Pharmacology and Department of Neuroscience, College of Medicine, The Ohio State University Wexner Medical Center, Columbus, OH 43210 USA; Department of Molecular and Cellular Medicine, Texas A&M University, College Station, TX 77483 USA; Department of Biology, New Mexico State University, Las Cruces, NM 88003 USA; Department of Biology, University of Louisiana, Lafayette, LA 70503 USA; University of Texas, Austin, TX 78712 USA; The Josephine Bay Paul Center for Comparative Molecular Biology and Evolution, The Marine Biological Laboratory, Woods Hole, MA 02543 USA; Systemix Institute, Redmond, WA 98053 USA

**Keywords:** Electric eel, Genome, Transcriptome, miRNA, Gene ontology

## Abstract

**Background:**

With its unique ability to produce high-voltage electric discharges in excess of 600 volts, the South American strong voltage electric eel (*Electrophorus electricus*) has played an important role in the history of science. Remarkably little is understood about the molecular nature of its electric organs.

**Results:**

We present an in-depth analysis of the genome of *E. electricus*, including the transcriptomes of eight mature tissues: brain, spinal cord, kidney, heart, skeletal muscle, Sachs’ electric organ, main electric organ, and Hunter’s electric organ. A gene set enrichment analysis based on gene ontology reveals enriched functions in all three electric organs related to transmembrane transport, androgen binding, and signaling. This study also represents the first analysis of miRNA in electric fish. It identified a number of miRNAs displaying electric organ-specific expression patterns, including one novel miRNA highly over-expressed in all three electric organs of *E. electricus*. All three electric organ tissues also express three conserved miRNAs that have been reported to inhibit muscle development in mammals, suggesting that miRNA-dependent regulation of gene expression might play an important role in specifying an electric organ identity from its muscle precursor. These miRNA data were supported using another complete miRNA profile from muscle and electric organ tissues of a second gymnotiform species.

**Conclusions:**

Our work on the *E. electricus* genome and eight tissue-specific gene expression profiles will greatly facilitate future research on determining the coding and regulatory sequences that specify the function, development, and evolution of electric organs. Moreover, these data and future studies will be informed by the first comprehensive analysis of miRNA expression in an electric fish presented here.

**Electronic supplementary material:**

The online version of this article (doi:10.1186/s12864-015-1288-8) contains supplementary material, which is available to authorized users.

## Background

The electric eel (*Electrophorus electricus*) is a freshwater teleost (order: Gymnotiformes) from South America, the only species identified to date within the genus *Electrophorus* [1]. Reaching more than seven feet in total length, *E. electricus* is most famous for its ability to generate strong voltage discharges (up to ~600 volts [2]) from electric organ (EO) tissues for use in predation and defense. Because of this remarkable ability*, E. electricus* has played a prominent role in the history of science – in physics, for early insights into the nature of electricity, and in biochemistry, as a rich source of tissue for extensive biochemical investigations of ion channels and pumps [3].

**Figure 1:**
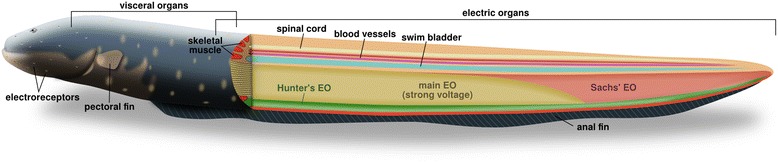
**Overview of electric eel anatomy.** Longitudinal section of *E. electricus* showing location and relative size of the three electric organs along with other anatomical features.

Over 700 species of electric fishes have been identified [1], the vast majority of which are capable of generating only weak electric organ discharges (EOD) for the purpose of navigation and communication. Like other members of Gymnotiformes, *E. electricus* produces weak EODs (mV-V scale) for navigation and communication. However, it is unique within Gymnotiformes in possessing three distinct EOs (most other Gymnotiformes have only one distinct organ, and some have additional accessory organs), and it is the only gymnotiform capable of a strong-voltage EOD. In *E. electricus*, strong EODs are produced from the main EO and the anterior two-thirds of the more ventrally-positioned Hunter’s EO; weak EODs are produced from the Sachs’ organ and the posterior one-third of the Hunter’s organ (Figure 1). All three EOs of *E. electricus* are derived developmentally from a germinal zone located on the ventral margin of the hypaxial musculature [4,5]. Interestingly, the ability to produce EODs is not limited to the Gymnotiformes; indeed, electric organs have evolved independently from skeletal muscle at least six times in fishes [4,6].

Despite the importance of electric fishes in the history of science, genomic, proteomic, and metabolomic approaches towards understanding the molecular nature of electrocytes (the single cell of the electric organ) have only lately been undertaken [7-16]. In a recent report from this consortium, we described a small group of protein-coding genes that showed similar patterns of expression in electric organs as compared to skeletal muscle from three distinct lineages in which the electrogenic phenotype evolved independently [13]. Included in this study was the first draft genome sequence of *E. electricus*, but a detailed analysis of gene content and tissue-specific expression in this electric fish species remained to be described. In this report, we describe the first comprehensive analysis of genes and multi-organ gene expression of *E. electricus*. Our gene set enrichment analysis using Gene Ontology terms found that genes highly expressed in EOs are enriched in functions pertaining to transmembrane transport, receptor signaling, and hormone binding. We performed the first analysis of microRNA (miRNA) expression in an electric fish and show that all three EOs in *E. electricus* express a unique repertoire of miRNAs, including a novel miRNA and three conserved miRNAs involved with muscle development inhibition in mammals. The results build a framework for comprehensively understanding the molecular nature of an electrocyte and provide a foundation for future work on electric organs in electric fish.

## Results

### *E. electricus* genome features

We used next-generation sequencing technologies to sequence and assemble the genome of *E. electricus* and the transcriptome of the three EOs and five other tissues: brain, spinal cord, heart, skeletal muscle, and kidney, as described previously [13] (Additional file 1: Table S1a). A set of 29,363 gene models representing an estimated 22,000 protein-coding genes was annotated from the genome and transcriptome. Comparison between the genomes of *E. electricus* and *Danio rerio*, the nearest related sequenced fish genome*,* showed considerable local synteny (i.e., *hox* genes, see Additional file 1: Figure S1a). The average intron size in *E. electricus* was similar to that of the other sequenced non-pufferfish teleosts and was ca. one-third that of *D. rerio* (Additional file 1: Table S2).

### *E. electricus* transcriptome analysis

**Figure 2:**
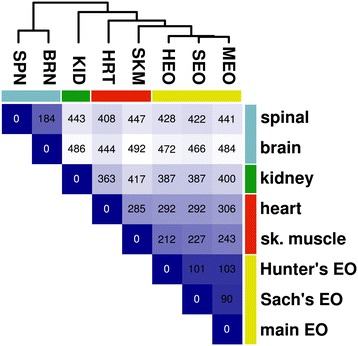
**Clustering of eight electric eel tissues by gene expression profile.** Gene expression values for the eight tissues were normalized, variance filtered, log_2_-transformed and median-centered as described previously [13]. Values shown are Euclidean distances based on ca. 6,000 genes passing the covariance filter, also indicated by blue shade (darker indicates shorter distance). Clustering was performed using complete linkage hierarchical clustering. Colored bars indicate a general grouping by tissue and cell type that is suggested by the data, with electric organ tissues (yellow) clustering most closely with skeletal and heart muscle (red). SPN = spinal cord; BRN = brain; KID = kidney; HRT = heart; SKM = skeletal muscle; HEO = Hunter’s EO; SEO = Sachs’ EO; MEO = main EO.

**Figure 3:**
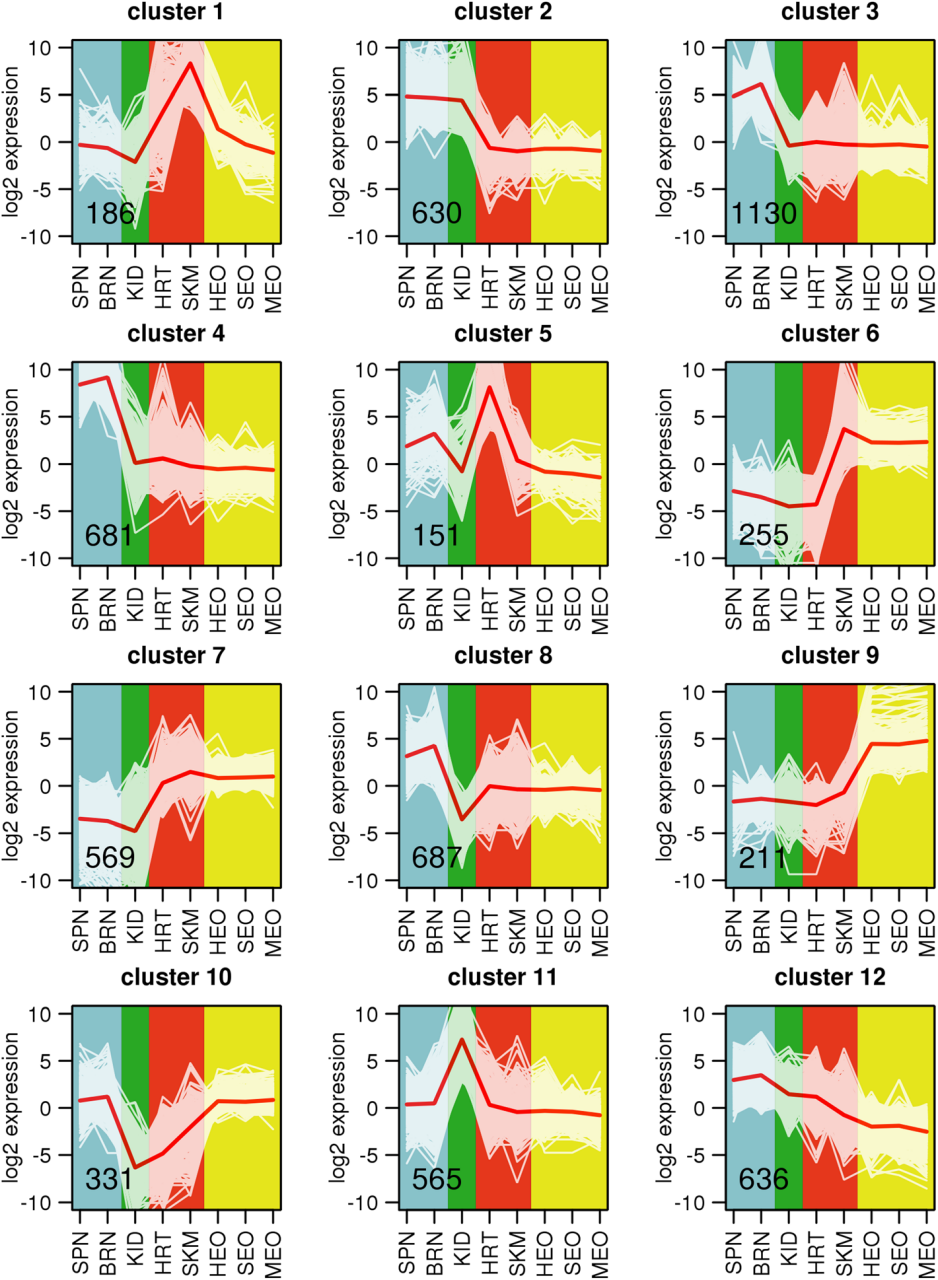
**Clustering of co-expressed genes in** ***E. electricus*** **.** Figure reproduced from [13]. A k-means clustering analysis (k = 12) was performed as previously described [13]. Values in lower-left indicate the number of genes in each cluster. White plot lines represent log_2_-transformed and median-centered expression of individual genes and red plot lines show median values for the cluster. Background shading indicates general categories of tissue/cell type. SPN = spinal cord; BRN = brain; KID = kidney; HRT = heart; SKM = skeletal muscle; HEO = Hunter’s EO; SEO = Sachs’ EO; MEO = main EO.

Our comparison of genes expressed in eight organs of *E. electricus* [13] showed that the mRNA expression profiles of electrocytes found in the three EOs (Hunter’s, Sachs’ and main) were distinct from all other cell types, with a greater similarity in gene expression to skeletal and heart muscle as compared to kidney, brain or spinal cord (Figure 2). This finding was consistent with the known myogenic origin of electrocytes in most species [4]. Variance filtering of the gene models predicted in our first computational annotation removed ~ 3/4 of the genes with low covariance among tissues. A subsequent k-means clustering (k = 12) revealed sets of tissue-specific co-transcriptionally regulated genes ([13] and Figure 3). Of particular interest were clusters 1, 6, 7, 9, and 10, which represented genes over-expressed only in EOs (cluster 9), genes over-expressed in skeletal and heart muscle (cluster 1), genes over-expressed in both skeletal muscle and EO (cluster 6), genes over-expressed in skeletal muscle, heart and EO (cluster 7), and genes over-expressed in brain, spinal cord and EO (cluster 10) (Figure 3). Clusters 6 and 7 represented a shared identity between electrocytes and myocytes, while clusters 1 and 9 represented sets of genes that were down- or up-regulated in electrocytes compared to myocytes and may hold clues to the unique structure and function of the EO.

In order to understand what functions were enriched and best characterized in our tissue-specific expression clusters, we performed a Gene Ontology (GO) enrichment analysis on each of the 12 tissue-specific clusters. This analysis revealed enriched functions that were consistent with expectations based on the tissues in the tissue-specific clusters (Additional file 2). For example, GABA-A receptor activity, ionotropic glutamate receptor activity, and extracellular-glutamate-gated ion channel activity appear enriched in clusters 3 and 4, both of which are gene clusters over-expressed in brain and spinal cord. In cluster 11 (kidney), enriched GO terms are consistent with fish kidney (fish kidneys are used not only for osmoregulation but also for hematopoiesis), including several GO terms involved with transmembrane transport as well as heme binding.

**Figure 4:**
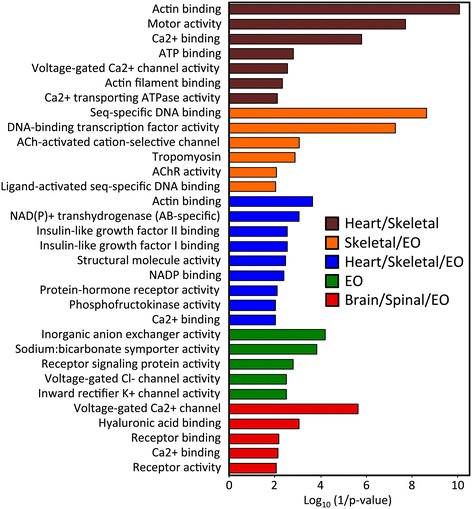
**Gene Ontology enrichment of genes over-expressed in muscle and electric organ of** ***E. electricus*** **.** Enrichment of GO terms in the “molecular function” ontology. Shown are enriched GO terms identified using topGO in cluster 1 (over-expressed in skeletal and heart muscle), 6 (over-expressed in skeletal muscle and EO), 7 (over-expressed in skeletal muscle, heart and EO), 9 (over-expressed only in EOs), and 10 (over-expressed in brain, spinal cord and EO) (p < 0.01).

GO analysis of cluster 9 (all EOs) showed an enrichment of GO terms involved with transmembrane transporting (Figure 4), while enriched GO terms of cluster 1 (skeletal muscle and heart) consisted of calcium-transporting ATPase activity, voltage-gated calcium channel activity, and calcium ion binding, highlighting the well-known role of Ca^2+^ in muscle contraction. In cluster 6 (all EOs and skeletal muscle), the most enriched GO terms involved the general category of transcriptional regulation, including sequence-specific DNA binding, ligand-activated sequence-specific DNA binding, and sequence-specific DNA binding transcription factor activity, as well as GO terms involved with acetylcholine receptor activity. In cluster 7 (all EOs, skeletal muscle, and heart), the enriched GO terms were involved in metabolism, such as NADP binding, NAD (P) + transhydrogenase activity, and phosphofructokinase activity, as well as insulin-like growth factor (IGF) I and II binding. In cluster 10 (all EOs, brain, and spinal cord), the enriched GO terms interestingly included voltage-gated Ca^2+^ activity and Ca^2+^ binding. Additionally, cluster 10 shows enrichment in receptor binding, receptor activity, and hyaluronic acid binding.

TopGO was further used to generate GO graphs for each of the five primary clusters of interest (1, 6, 7, 9, & 10) using enriched GO terms (p-value < 0.05) as input (Additional file 1: Figure S2). The findings were consistent with those of Figure 4, but additionally highlight highly represented categories unique to each cluster. Especially informative are the GO graphs generated for clusters 6, 7, and 9. The GO graph generated for the enriched GO terms in cluster 6 (skeletal muscle and electrocytes) has a large, highly represented group broadly characterized as metabolism (7 out of 19 total terms). Additionally, broad categories including actin/tropomyosin binding are also over-represented in cluster 6; this is intriguing, as the electrocytes have lost their contractile machinery. Also, skeletal muscle cells and electrocytes are activated by acetylcholine, and this is reflected in the graph, including the GO terms for acetylcholine-activated cation-selective channel activity and acetylcholine receptor activity.

Similar to the results from cluster 6, the GO graph generated for the enriched terms in cluster 7 (skeletal muscle, heart, and all EOs) had a highly represented, broad category of metabolism (7 out of 20 total terms). Finally, the cluster 9 (all EOs) GO graph has a highly represented group characterized as transmembrane transport (7 of 16 total terms). This group includes GO terms such as voltage-gated sodium channel activity and inward rectifier K^+^ channel activity, which are directly involved in electric organ discharge (EOD). Cluster 9 also includes GO terms involved in hormone/androgen binding; this GO term is physiologically relevant as EOD has been shown to be regulated in part by the presence of sex hormones [17].

### *Hoxc* cluster expression in EO

One surprise arising from the 8-tissue profiling was the elevated expression of *hoxc10a*, *hoxc11a*, *hoxc12a* and *hoxc13a* genes in all three electric organs (Additional file 1: Figure S1b). Hox family members are well-known components of the regulatory machinery that specifies the anterior-posterior body axis of animals, and in many cases the spatial expression patterns of *hox* genes within tandem modules on the genome have been observed to correlate with spatial distribution of expression along that axis [18]. We observed the same set of *hox* genes (*hoxc10a*, *hoxc11a*, *hoxc12a*, *hoxc13a*) from the *hoxca* cluster to be over-expressed in two other Gymnotiformes (*Eigenmannia virescens* and to a lesser extent *Sternopygus macrurus*) and one mormyrid (*Brienomyrus brachyistius*) as well (Additional file 1: Figure S1c and [13]); interestingly, these *hoxc* genes are not highly expressed in the EO of the electric catfish *Malapterurus electricus* (data not shown). Jawed vertebrates have four paralogous *hox* cluster genes (*hoxa*, *hoxb*, *hoxc*, *hoxd*), among which only the *hoxc* cluster was shown to be dispensable for body plan development. The entire cluster was lost in Elasmobranch fishes and its deletion in mice caused only minor transformations of axial identity [19-22]. Whether these are retained in adults to specify the predominant posterior location of the electrocytes in Gymnotiformes and mormyrids (but not electric catfish) or have another function is not known. Our observation raises the possibility of neofunctionalization of posterior *hoxca* genes in some species of electric fishes.

### Analysis of binding sites for highly upregulated transcription factors in EO

A significant future goal is to understand the mechanisms underlying EO development and maintenance. As a step toward that goal, a plausible hypothesis is that transcription factors highly upregulated in EO regulate distinctive characteristics of EO. Based on this hypothesis, candidates for this set of important transcription factors were identified by their high expression ratios (>7-fold) in EO compared to skeletal muscle (Additional file 3, A). Within this set, a subset of transcription factors were particularly promising candidates because they were also highly expressed in EO compared to all five non-EO tissues including skeletal muscle: *egr3* (early growth response 3), *six2a* (sine oculis-related homeobox 2a), *hoxc11a* (homeo box C11a), *foxj3* (forkhead box J3), *ar* (androgen receptor), *pou3f1* (POU class 3 homeobox 1), *lbx2* (ladybird homeobox homolog 2), and *hoxc10a* (homeo box C10a).

One possible explanation for how cluster 9 genes become upregulated in EO is enrichment of binding sites within their promoters for one or more transcription factors that are themselves highly expressed in EO. To test this hypothesis, we examined putative promoter regions within cluster 9 genes for binding sites of 21 transcription factors highly expressed in EO relative to skeletal muscle, using 2984 randomly-sampled genes as a background control (Additional file 3). Their DNA binding sites were frequently found in putative promoter regions of cluster 9 genes (see Additional file 3, B for examples). From testing the density of binding sites in promoters of all cluster 9 genes as a group compared with background controls, the smallest p-values suggesting binding site enrichment were found for transcription factors *prrx1b* (paired related homeobox 1b; *p* = 0.006) and *lbx2* (ladybird homeobox homolog 2; *p* = 0.049) (Additional file 3, C). From the subset of 51 of the cluster 9 genes most highly expressed in EO relative to skeletal muscle (Additional file 3, D), the smallest p-values suggesting enrichment were found for transcription factors *prrx1b* (*p* = 0.02) and *emx2* (empty spiracles homeobox 2; *p* = 0.047). We then compared the number of occurrences of each transcription factor binding site in the promoters of the 51 most highly expressed cluster 9 genes individually against our background control and found p-values smaller than 0.005 for *emx2*, *lbx2*, *pou3f1* (POU class 3 homeobox 1), *prrx1b*, and *sox12* (SRY-box 12) (Additional file 3, E). These results suggest particular highly upregulated transcription factors that might contribute to upregulation of cluster 9 genes in EO through selective enrichment of their DNA binding sites and are possible targets for further study. It is important to note, however, that none of these p-values remained significant at a 5% FDR after multiple testing correction, and that the magnitude of change compared to background for the genes/binding sites discussed was generally relatively small.

### Parallel evolution in the Kir2 channel and the Na+/K + −ATPase

It has been reported that some electrocyte-specific ion channels involved in generating the electric discharge appear to be evolving at a higher than expected rate in electric fishes (see [23,24] for a discussion of the Na_V_1.4a sodium channels in electrocytes). Two teleost-specific members (*kcnj2b*, *kcnj12b*) of the inward rectifying K^+^ channel (Kir2) family are abundant in *E. electricus* electrocytes. A hallmark of Kir2 channels is a highly conserved aspartate residue at the inner mouth of the channel that binds Mg^2+^ and polyamines and plugs the channel at depolarizing voltages imparting rectification [25,26]. In the non-rectifying members of the Kir family, there is an asparagine residue instead at that site. Within the channels encoded by the gymnotiform *kcnj2b* and *kcnj12b*, both have an asparagine at the Mg^2+^ binding site, suggesting that the gymnotiform electrocyte has a unique intracellular environment. In addition, the α2 isoform of the sodium pump, which is highly over-expressed in the electrocyte, shows an amino acid substitution at a conserved site (Additional file 1: Figure S3). In an interesting case of parallel evolution, the same substitution occurs in squid, although there it is due to RNA editing rather than a permanent change in the codon. This amino acid change is thought to enhance sodium transport [27].

### Reduced vision and loss of opsin genes in *E. electricus*

Diurnally active teleost fishes generally have four physiologically distinct cone types in their retinae; one with long, one with medium, and two with short wavelength-sensitive opsins [28]. In contrast, *E. electricus* is nocturnally active and often lives in muddy rivers and streams where the ambient light is strong in longer wavelengths [29,30]*.* We searched the *E. electricus* genome for opsin genes; interestingly, we found only long (red), and medium (green) but no short-wave sensitive (blue and violet) cone opsin genes (Additional file 1: Figure S4). Although possible, it is unlikely that this pattern is due to incomplete sequencing coverage of the genome as we also recovered a gene for the rod photopigment rhodopsin and numerous other teleost non-photopigment opsins such as melanopsin. We hypothesized that the lack of short-wave sensitive cone opsin genes may be shared with other species that live in similarly muddy and murky conditions. Indeed, when we probed an EST database of a species in a sister group of Gymnotiformes, a non-electrogenic catfish [31], we observed the same cone opsin profile as *E. electricus*.

### MiRNA analysis: novel sequences and EO-specific expression

**Figure 5:**
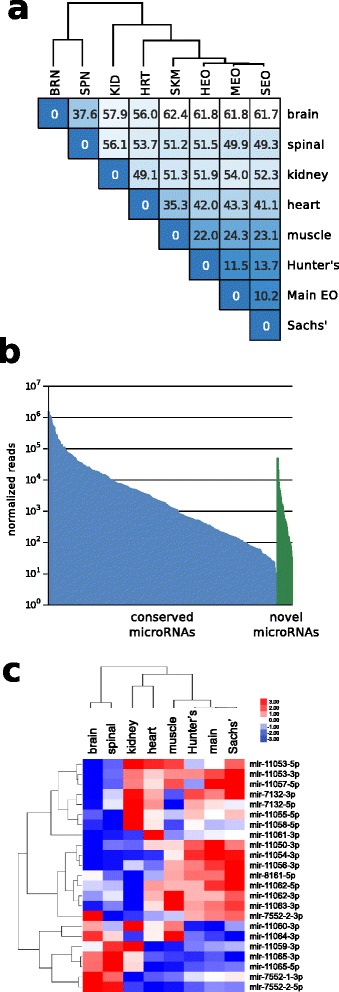
**Known and novel miRNA genes. (a)** MiRNA expression classifies *E. electricus* tissues. Tissue distance matrix based on miRNA expression. MiRNA expression values for 8 tissues were filtered, normalized and log_2_ transformed as described in Methods. Values shown are Euclidean distances. Tree is derived from complete linkage hierarchical clustering. **(b)** Normalized sequencing read counts for conserved and novel *E. electricus* miRNAs. **(c)** Heatmap and complete linkage hierarchical clustering of novel miRNA log_2_-transformed and median-centered expression values in *E. electricus* tissues demonstrates tissue-specific expression patterns. Log2 values are clamped between −3 and +3.

MiRNAs are an evolutionarily ancient class of small non-coding RNAs that regulate many gene networks during animal development [32]. MiRNA composition and expression levels have been used as a molecular taxonomy approach for categorization of tissue types, description of cellular physiological states and even classification of disease states [33]. It has also been suggested that expansion of miRNA families has played a central role in the remarkable morphological complexity among vertebrates [34]. To investigate the potential role of miRNAs in electrocyte phenotype and function, we isolated and sequenced small RNAs from the spinal cord, brain, heart, skeletal muscle, kidney, and all three EOs of *E. electricus*. We identified 294 conserved miRNAs belonging to 119 miRNA families expressed in one or more of the eight tissues [35-38]. We also identified 18 novel miRNAs from the set of unmatched reads with perfect matches to the *E. electricus* genome (Figure 5c). As shown in other organisms, conserved miRNAs tend to be more robustly expressed than species-specific miRNAs [39-41] (Figure 5b). However, all novel miRNAs found in *E. electricus* showed tissue-specific expression patterns, suggesting that they may serve specific functions in *E. electricus*.

**Figure 6:**
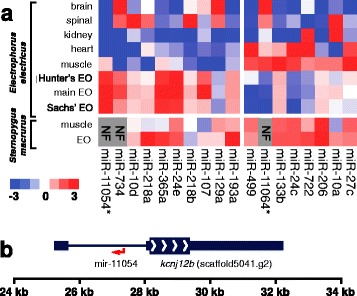
**Electrocyte-specific microRNA expression. (a)** Heatmap of miRNA expression in *E. electricus* and *S. macrurus.* Values are log_2_-transformed and median-centered values of tissue-specific expression for each miRNA, such that blue indicates under-expression and red indicates over-expression relative to the median. The miRNAs shown are limited to those with >4-fold increased or decreased expression in *E. electricus* electric organs compared to skeletal muscle. Log_2_ values are clamped between −3 and +3. Asterisks indicate novel *E. electricus* miRNAs. **(b)** Schematic diagram of the *kcnj12b* gene locus and novel electro-miR mir-11054 on scaffold5041 of the *E. electricus* genome. Thin boxes are UTRs, the thick box with white directional arrows is the coding sequence, and the thin line is an intron. The red arrow on the antisense strand indicates the location of the novel mir-11054 microRNA.

To investigate the role of miRNAs in the EO, we performed hierarchical clustering of miRNA expression across the eight *E. electricus* tissues. MiRNA expression patterns clustered nervous tissue from the brain and spinal cord separately from cardiac muscle, skeletal muscle, and EOs, and clustered EOs more closely with skeletal muscle than cardiac muscle (Figure 5a). Indeed, from the 312 total miRNAs identified (294 conserved and 18 novel) only 18 showed high differential expression between skeletal muscle and the three EOs (Figure 6a). Conserved miRNAs with lower expression in EO than in muscles have annotated roles in muscle development and differentiation in other organisms [42-44]. Three of these miRNAs are muscle-specific, also called “myomiRs” (miR-133, miR-206, and miR-499), because they play critical roles in muscle development and function. In contrast, miRNAs with higher expression in EO than in muscle include multiple miRNAs with roles including inhibition of muscle differentiation (miR-193, miR-218, and miR-365) [45,46].

The most upregulated miRNA in EO compared to muscle is the novel miRNA mir-11054 (Figure 6a). This electrocyte specific “electromiR” was 30-fold higher in all three EOs compared to skeletal muscle and was not detected in brain, spinal cord, kidney, or heart tissues of *E. electricus*. Notably, mir-11054 is expressed from a locus that is important to electrocyte function, an intron of the inward-rectifier K^+^ channel gene *kcnj12b* that is abundant in electrocytes. Though it is expressed from a region overlapping the *kcnj12b* gene, mir-11054 is expressed in the antisense direction from an intronic sequence that is unique to *E. electricus* (Figure 6b). This “electromiR” mir-11054 has no known homologue in any fish or mammal to date.

To further probe the importance of the differentially expressed miRNAs in EO function, we sequenced miRNA libraries from the EO and skeletal muscle of a second electric fish, *S. macrurus*. This comparison revealed interesting differences and similarities between the two Gymnotiformes species (Figure 6a). ElectromiR mir-11054, which is highly transcribed in *E. electricus* EO from the K+ channel intron, is not detected in *S. macrurus*, suggesting that it may be specific to *E. electricus*. In addition, our results indicate that, in contrast to the downregulation observed in *E. electricus* EO, *S. macrurus* EO expresses most myo-miRs at levels similar to those found in skeletal muscle. The continued expression of myomiRs in *S. macrurus* EO is consistent with a more ‘muscle-like’ phenotype in this fish [47]. In contrast to myomiRs, miRNAs with annotated roles in inhibiting muscle differentiation are commonly upregulated in the EO of *E. electricus* and *S. macrurus*. These miRNAs include miR-218a, which inhibits cardiac muscle development [48], and the bicistronically-encoded miR-365a and miR-193a, which inhibit skeletal muscle development [49].

## Discussion

The analyses presented here describe molecular comparison of *E. electricus* electric organs to muscle and other tissues and build off of this consortium’s previous work exploring the convergent evolution of electric organs in independent fish lineages [13]. Analyses of the *E. electricus* genome identified a number of interesting characteristics. The *E. electricus* genome is approximately ~700 Mb in size, which is roughly half of that of *D. rerio*. By comparing gene models across available fish genomes, we found that *E. electricus* intron lengths were about one third that of *D. rerio* (Additional file 1: Table S2), which likely is a significant contributing factor to the difference in genome sizes among the sequenced non-pufferfish teleosts and *D. rerio*. We also found a number of genomic changes that contribute to the adaptation of *E. electricus* environment and physiology. For example, within the α2 subunit of the sodium pump, which is highly abundant in EO, there is an amino acid substitution that has been demonstrated previously to occur in squid and is thought to enhance sodium transport [27] (Additional file 1: Figure S3). Given the important role of sodium transport in EOD, this substitution may be important for electrocytes to rapidly relieve high internal [Na^+^] after depolarization. Of interest, this finding seems to be specific to *E. electricus*, as *E. virescens* and *S. macrurus* do not share this substitution. As another example, *E. electricus* lives in muddy rivers and streams where short wavelength light is more easily filtered out [29,30]. We were unable to identify short-wave light sensitive opsin genes in the *E. electricus* genome despite being able to identify long and medium-wave opsin genes. Upon examination of the non-electrogenic catfish that resides in a similar environment [31], we observed a similar opsin profile. Thus, the loss of short wavelength-sensitive opsins is likely adaptive as it allows for a greater number of photoreceptors with opsins in the most useful portions of the visual spectrum. The absence of short wavelength-sensitive opsins has also been reported in most mammals living under dim light conditions or which, like bats and cetaceans, utilize other specialized sensory systems (e.g. echolocation) [50,51]. It is interesting to note that catfish took a different path in adapting to nocturnal living by developing taste buds all over their body to ‘taste’ the environment [52], much as electric fish sense their surroundings using electroreceptors.

One main goal of our research was to characterize the genes expressed in each of our eight tissues, with particular focus on understanding the unique repertoire of genes expressed in EO compared to skeletal muscle. To that end, we clustered our genes by similarity of expression (Figure 3), and within the resulting clusters employed GO term enrichment techniques to characterize enriched functions (Figures 4 and Additional file 1: Figure S2). The EO-specific cluster (cluster 9) showed unique aspects of EO that are not shared with muscle (the tissue type from which it is developmentally derived). Within this cluster, we found an abundance of genes with “transmembrane transporting” function and a lack of “cytoskeletal binding” and other contraction-related terms. This result reflected the tradeoff between contractility-related physiological function and increased electrogenic output. Conversely, we identified an enrichment of metabolism-related genes within all myogenic tissues (skeletal muscle, heart, and EO), which suggested that the basic metabolic processes in muscle are retained in electrocytes from myogenic precursors. Recent efforts from this consortium have identified IGF signaling as an important element in the independent evolution of electric organs [13]. Interestingly, our functional enrichment analysis revealed enrichment for IGF binding in myogenic tissues (skeletal muscle, heart, and EO). Looking into this further, although IGF binding was enriched both in muscles and EO of *E. electricus* in the analyses presented here, only the *regulators* of this signaling pathway were up-regulated in electric organs compared to skeletal muscle in *E. electricus*, in other Gymnotiformes, and in two other lineages of fishes with independently evolved electric organs [13], indicating IGF signaling likely has a specialized role in EO over muscle. As we previously reported [13], our hypothesis is that IGF signaling contributes to the increase in cell size of electrocytes over muscle fibers.

Within the cluster of genes co-expressed in EO, brain, and spinal cord (cluster 10) we found enrichment for terms pertaining to Ca^2+^ transport and binding. This may highlight a gain of an additional Ca^2+^ function in EO that utilizes genes expressed typically in brain and spinal cord despite no need for Ca^2+^ for contraction (indeed, EO has turned down expression of genes relating to Ca^2+^ function). However, we cannot rule out the possibility that nerve contamination in EO tissue (which is highly innervated) contributed to this result, a possibility that would require additional experimentation to rule out.

A crucial step in our greater quest to elucidate the mechanisms by which electric organs have evolved is understanding the underlying principles of how genes are regulated in EO compared to muscle. Given that several transcription factors are highly upregulated in EO, we hypothesized that there may be enrichment of these transcription factor binding sites in the promoters of genes that are also highly expressed in EO. However, our attempts at identifying enriched binding motifs within the promoters of genes highly expressed in EO (cluster 9 genes) failed to identify a “smoking gun”; thus, it is reasonable to suspect that other mechanisms beyond enrichment of DNA binding sites for highly expressed transcription factors in EO might be responsible for regulation of cluster 9 genes. First, binding sites for transcription factors that are not upregulated might be enriched in cluster 9 genes and lead to transcriptional upregulation in EO. This mechanism, however, does not explain the restriction of cluster 9 gene upregulation to only EO. As an additional mechanism, factors regulating gene transcription, including chromatin state, might differentially affect availability of these binding sites in cluster 9 genes compared to availability in genes of other clusters. The upregulation of seven transcription factor genes (*hoxc10a*, *hoxc11a*, *hoxc12a*, *hoxc13a*, *six2a*, *sox11b*, and *mef2b*) in EO from four electric fishes (*E. electricus*, *S. macrurus*, *E. virescens*, and *B. brachyistius*) [13] suggests their particular importance in EO identity. The lack of enrichment of binding sites for these seven, however, suggests that other mechanisms beyond transcription factor upregulation are involved in expression of cluster 9 genes—an exciting area for future research studies.

MiRNAs play important roles in regulating gene networks throughout animal development [32]. We aimed to characterize miRNA expression in our eight tissues of interest, with particular focus on muscle and EO and with the goal to determine whether there was a potential role of miRNA in EO development or maintenance. Our analysis revealed nearly 300 conserved miRNAs with known functions and 18 novel miRNAs; these novel miRNAs showed tissue-specific expression patterns which indicated they may be serving tissue-specific functions in *E. electricus*. Of particular interest were the 18 miRNAs that showed high differential expression among skeletal muscle and three EOs (Figure 6a), and, to gauge whether our findings were *E. electricus*-specific or shared among Gymnotiformes, we also performed miRNA sequencing and expression analysis on *S. macrurus*. Of particular note were three conserved muscle-specific miRNAs that were highly expressed in EO relative to skeletal muscle (miR-193, miR-218 and miR-365) in both Gymnotiformes tested; these miRNA have known roles in inhibiting muscle differentiation [45,46]. Interestingly, we identified a novel “electromiR” abundantly expressed in *E. electricus* EO but not identified in *S. macrurus* EO, implying that this novel miRNA arose for *E. electricus*-specific electrocyte development and function (Figure 6b). The upregulation of conserved miRNAs with known roles in blocking muscle development in the EO of both *E. electricus* and *S. macrurus* provides evidence that miRNAs are part of a common toolkit involved in the development and maintenance of the electrocyte phenotype in Gymnotiformes. Uncovering the functional role of miRNAs that are uniquely expressed in *E. electrophorus* EOs could shed light on the molecular mechanisms involved in the modification of the muscle program to give rise to such a specialized tissue as the EO.

## Conclusions

We describe here an analysis of the first sequenced genome of an electric fish (*E. electricus*) and of mRNA and miRNA libraries from eight organs including the three electric organs. This study, which builds upon previous work from this group focusing on shared protein-coding gene expression patterns between EO and skeletal muscle in multiple independent lineages of electric fish [13], provides a focused and thorough examination of both novel genomic characteristics as well as protein- and microRNA-encoding gene expression patterns and gene set enrichment in a panel of diverse organs from the strong voltage electric eel. Genes expressed in electric organs were enriched for functions involving transmembrane transport, whereas skeletal muscle showed enrichment for contraction-related functions, reflecting the specialization of electrocytes for electrogenesis over contraction. Gene expression shared between skeletal muscle and electric organs had functional enrichment for genes relating to metabolism, suggesting that metabolic characteristics of each cell type are similar even though the chemical energy is transduced to a different degree in terms of mechanical versus electrical energy. The first comprehensive analysis of miRNA expression in electric fish identified three conserved miRNAs that have known roles in inhibiting muscle development as highly expressed in electrocytes, suggesting that miRNAs may be playing an important role in electrocyte development and maintenance. Interestingly, one of the 18 novel miRNAs identified was highly specific to EO and was transcribed from the reverse strand of an intron within the potassium channel that is also highly expressed in EO. Future studies will build from the work presented here to understand more deeply the function and evolution of genes expressed in electric organs, including the molecular and evolutionary distinction of the strong voltage electric organ unique to *E. electricus* in the Gymnotiformes lineage.

## Methods

### Analysis of gene ontology enrichment in clusters with tissue-specific expression

*E. electricus* gene models that were described previously [13] as being short fragments of whole genes and labeled “split” or “split_scaff” were filtered out for this analysis. Gene Ontology (GO) terms assigned to each *D. rerio* gene were downloaded from Ensembl (release version 71). GO terms were mapped back to all possible *E. electricus* genes using their *D. rerio* assignments. A total of 13,647 *E. electricu*s genes remained in the analysis after these steps. In order to prevent the presence of a single GO term in one of our gene clusters from coming up as significant, we filtered out all GO terms from the tissue-specific clusters that were present less than two times. Enriched GO terms in each of the 12 tissue-specific clusters were identified by using the “elim” method of topGO [53], with a minimum node size of 3. The elim method of topGO attempts to eliminate some of the local dependencies inherent to the GO graph structure by removing genes mapped to higher level GO terms, as to emphasize lower level (more specific) GO terms. We used the Fisher’s exact test to identify significantly enriched GO terms with a p-value < 0.01. For clusters 1, 6, 7, 9, and 10, we used topGO to create the GO graph resulting from these enriched terms.

### MiRNA sequencing and analysis

We measured miRNA expression in eight tissues (brain, spinal cord, heart, skeletal muscle, Sachs’ electric organ, main electric organ, Hunter’s electric organ, and kidney) of *E. electricus*.

#### Small RNA library preparation and sequencing in E. electricus

One (1) μg total RNA from each tissue (isolated as described above for mRNA sequencing) was used to prepare small RNA sequencing libraries using an Illumina TruSeq Small RNA Library Preparation Kit according to the manufacturer’s instructions (Illumina, Inc., San Diego, CA) with one modification: cDNA was size-selected on an agarose gel in the 140–300 bp range. Each of seven tissues (brain, spinal cord, heart, muscle, Sachs’, main, and Hunter’s EO) was labeled with a unique index using PCR indexing primers. The indexed small RNA libraries were pooled and sequenced on a single lane of HiSeq (1x100bp) at the same time as the corresponding mRNA was sequenced in our previous study ([13]). The kidney small RNA followed an identical protocol, except that it was done at a later date and was not indexed to be pooled with other samples. The linker sequences were removed from all libraries, and sets of trimmed reads of length 20, 21, 22 and 23 were compiled.

#### Identification of conserved and novel miRNA genes in E. electricus

Conserved *E. electricus* miRNA orthologs were identified by BLASTN comparison of expressed sequences from small RNA libraries to sequences from the Rfam database [54]. A set of potential novel miRNAs were generated by removing matches to conserved miRNAs, as well as contaminating small RNAs matching to a database of rRNA, snoRNAs, snRNAs, and mitochondrial rRNA and tRNAs derived from *Danio rerio*, *Tetraodon nigroviridis*, and *Takifugu rubripes* sequences in the Ensembl database [55]*.* To identify genomic precursors for conserved and novel miRNAs, sequences were aligned to the *E. electricus* genome, and perfect matches together with 140 bp of flanking sequence were retrieved. Secondary RNA structure was predicted using RNAFold from the Vienna package [56], and pre-miRNA hairpin structures were identified by the methods of [35]. MiRNAs were named according to their ortholog, and novel miRNAs were given unique names by miRBase [37].

#### Expression profiling and clustering

For expression analysis, small RNA libraries were matched to conserved and novel hairpin precursors in *E. electricus*, using a relaxed standard which allowed up to two base pair mismatches between sequences to account for known processes which add non-template nucleotides to the 3′ end of miRNAs [57], and for sequencing errors. Expression of 3p and 5p products was identified and calculated separately. Small RNA reads were adapter trimmed and filtered to 20–23 nucleotides in length. From this, 93% of small RNA reads from *E. electricus* tissue matched conserved miRNA families found in the *E. electricus* genome. Small miRNA sequences from *S. macrurus* were matched to precursors from *E. electricus* because of the lack of availability of a *S. macrurus* genome. MiRNA read counts for each tissue profiled in *E. electricus* and *S. macrurus* were first normalized by correction for the median number of total reads, followed by linear normalization (Additional file 4). MiRNAs which did not have at least 16 normalized reads in at least one tissue were discarded from further analysis. Complete linkage clustering of tissue-specific miRNA expression using the Pearson correlation distance (Figure 5a) was performed using Genepattern [58].

#### Analysis of transcription factor binding sites in cluster 9 genes

Searching the putative promoter sequences with MatInspector (Genomatix) with default settings for core and matrix similarity identified potential binding sites for transcription factors in promoter regions. A promoter was defined as the region 2 kilobases upstream from the transcription start site of a gene as determined by AUGUSTUS gene modeling. For statistical analysis, the number of binding sites for a transcription factor in putative promoters of cluster 9 genes as a group or as single genes was compared to the expected number. The expected number was derived from the binding sites in putative promoters of a set of 2984 randomly selected genes from all clusters. The p-value of a comparison was defined as the cumulative probability from a binomial distribution of the expected number of binding sites greater than or equal to the number of binding sites identified with MatInspector in cluster 9 genes. The p-values reported were not corrected for the number of comparisons.

The DNA binding properties of 21 highly upregulated *E. electricus* transcription factors selected for analysis with MatInspector were assumed to be identical to the binding properties of the homologous vertebrate transcription factors as described in MatBase (Genomatix). The relevance of DNA binding properties in MatBase to the properties of these *E. electricus* transcription factors has not been experimentally determined. Amino acid identities greater than 88% and averaging 96% for the DNA binding domains in these *E. electricus* transcription factors compared with mouse homologs supports the use of the MatInspector database for the analysis.

### Availability of supporting data

Whole genome sequence and annotation of *E. electricus* is available at http://efishgenomics.zoology.msu.edu together with BLAST and genome browsing capability. All raw sequencing reads are available in the National Center for Biotechnology Information Short Read Archive under BioProject IDs for the *E. electricus* genome (PRJNA249073) and transcriptome sequences for mRNA and miRNA analyses (PRJNA248545). All E. electricus miRNAs are available at miRBase. For gene and transcript expression analysis, see [13].

### Animal ethics statement

The University of Wisconsin Madison is accredited by the American Association of Laboratory Animal Care. The protocol governing the animal care and usage of *E. electricus* for this study was approved by the University of Wisconsin Animal Care and Use Committee (Protocol Number M01657). All procedures on *S. macrurus* followed the American Physiological Society Animal Care Guidelines, and were approved by the Institutional Animal Care and Use Committee at the New Mexico State University, NM (IACUC protocol 2014–044).

## Additional files

Additional file 1: Figure S1. Hox clusters and their expression patterns. **Figure S2.** Gene ontology enrichment in co-expressed genes of clusters 1, 6, 7, 9, and 10. **Figure S3.** Shared amino acid substitution in an abundant sodium pump of gymnotiform electrocytes. **Figure S4.** Phylogeny of opsin genes. **Table S1.** Summary of sequencing library depths. **Table S2.** Comparison of gene number and structure across species. Additional file 2:
**Functional enrichment analysis of tissue-specific clusters.** Table of enriched GO terms identified using elim method from topGO. Additional file 3:
**Analysis of transcription factor binding sites in cluster 9 genes.** Combined table of all promoter analysis results. (a) E. electricus transcription factors providing binding sites for the analysis of promoter regions of highly expressed genes in cluster 9. (b) Transcription factor binding sites on promoters of genes in cluster 9 with highest relative expression in EO compared to expression in skeletal muscle. (c) Density of binding sites of significant transcription factors for cluster 9 genes versus all E. electricus genes. (d) Density of binding sites of significant transcription factors for 51 highly expressed genes in cluster 9 compared to all E. electricus genes. (e) Probability of observed versus expected number of transcription factor binding sites in promoter regions of 51 highly upregulated genes in cluster 9. Additional file 4:
**MiRNA expression in electric fishes.** Table of normalized miRNA expression values in *E. electricus* and *S. macrurus*.

## Abbreviations

EO: Electric organ
EOD: Electric organ discharge
GO: Gene ontology
