# Supplementary material for: Unique patterns of transcript and miRNA expression in the South American strong voltage electric eel (Electrophorus electricus)
Source: BMC Genomics. 2015 Mar 26;16(1):243. doi: 10.1186/s12864-015-1288-8 (PMC4393597; doi:10.1186/s12864-015-1288-8)
Supplement: Additional file 1: Figure S1. — Hox clusters and their expression patterns. Figure S2. Gene ontology enrichment in co-expressed genes of clusters 1, 6, 7, 9, and 10. Figure S3. Shared amino acid substitution in an abundant sodium pump of gymnotiform electrocytes. Figure S4. Phylogeny of opsin genes. Table S1. Summary of sequencing library depths. Table S2. Comparison of gene number and structure across species. [file 12864_2015_1288_MOESM1_ESM.pdf]

Supplemental Figure Legends

Figure S1: Hox clusters and their expression patterns. (a) The arrangement of *hox* genes in *E. electricus* scaffolds relative to the *Danio* genome demonstrates both the completeness of the *E. electricus* genome sequence as well as the synteny between *E. electricus* and *D. rerio*. Gene pairs were identified based on two-way BLAST. Black arrows show the hox genes. Hollow white arrows show intervening gene models in *E. electricus* suggesting possible artifacts of the automated annotation pipeline. Hollow white boxes indicate lack of a predicted gene model but homology to the genome sequence at that location. Red box indicates a gene model which is present but which required manual adjustment of the gene model. Gray boxes indicate *D. rerio* chromosome and *E. electricus* scaffold on which the genes are located. (b) Expression of *hoxc-a* cluster in eight tissues of *E. electricus*. Normalized read counts were $\log_2$-transformed and plotted against gene location on the chromosome. (c) Subset of *hoxc-a* cluster genes overexpressed in electric organs of three Gymnotiformes and a mormyroid electric fish. Colors indicate $\log_2$ fold change between electric organ (as indicated) and skeletal muscle. Values were clamped at −4 and +4.

Figure S2: Gene ontology enrichment in co-expressed genes of clusters 1, 6, 7, 9, and 10. Enriched GO terms identified using "elim" method of topGO, and a minimum node-size of 3. All GO terms present only once were removed prior to analysis. GO graphs generated from all enriched terms (p-value < 0.05 by Fisher's exact test, represented as rectangular nodes in graph) identified in each of the four EO/muscle-containing clusters. The coloration of the enriched nodes indicates p-value size, such that the smallest p-values appear dark red, and the largest p-values appear pale yellow (those closest to the p-value cutoff of 0.05). (A) Graph of GO term topology generated from the 14 enriched GO terms in cluster 1. (B) Graph of GO term topology generated from the 19 enriched GO terms in cluster 6. (C) Graph of GO term topology generated from the 20 enriched GO terms in cluster 7. (D) Graph of GO term topology generated from the 16 enriched GO terms in cluster 9. (E) Graph of GO term topology generated from the 18 enriched GO terms in cluster 10.

Figure S3: Shared amino acid substitution in an abundant sodium pump of gymnotiform electrocytes. The α2 isoform of the sodium pump, which is highly over-expressed in the electrocyte, shows an amino acid substitution at a conserved site. (a) This substitution (red V) is present in *E. electricus*, *E. virescens* and *S. macrurus*, suggesting that it may have occurred at the origin of Gymnotiformes. It does not occur in the mormyrid species we have studied. In an interesting case of parallel evolution, the same substitution occurs in squid. (b) Illustration indicating approximate site of amino acid substitution (yellow asterisk) within the protein topology. (c) In squid the changed amino acid is due to RNA editing rather than a permanent change in the codon. This amino acid change is thought to cause enhanced sodium transport [27].

Figure S4: Phylogeny of opsin genes. Phylogenetic comparisons were made between multiple related fishes for long wavelength, medium wavelength, and short wavelength

opsin genes and rhodopsin gene. Both short wavelength opsins were missing from the *E. electricus* genome.

Table S1: Summary of sequencing library depths. Read counts for (a) *E. electricus* mRNA sequencing reads used in this study and in the previous study by this consortium [13] (b) *E. electricus* miRNA sequencing reads used in this study, and (c) *S. macrurus* miRNA sequencing reads used in this study.

Table S2: Comparison of gene number and structure across species. All *E. electricus* protein-coding genes were used in exon and intron length calculations (23,736 genes). Fragmented *E. electricus* gene models were removed prior to calculating coding sequence (CDS) length, transcript length, and untranslated region (UTR) lengths (19,039 genes analyzed). Gene statistics for other species were generated for protein-coding genes from whole-genome .gtf files downloaded from Ensembl (release version 70).

Figure S1

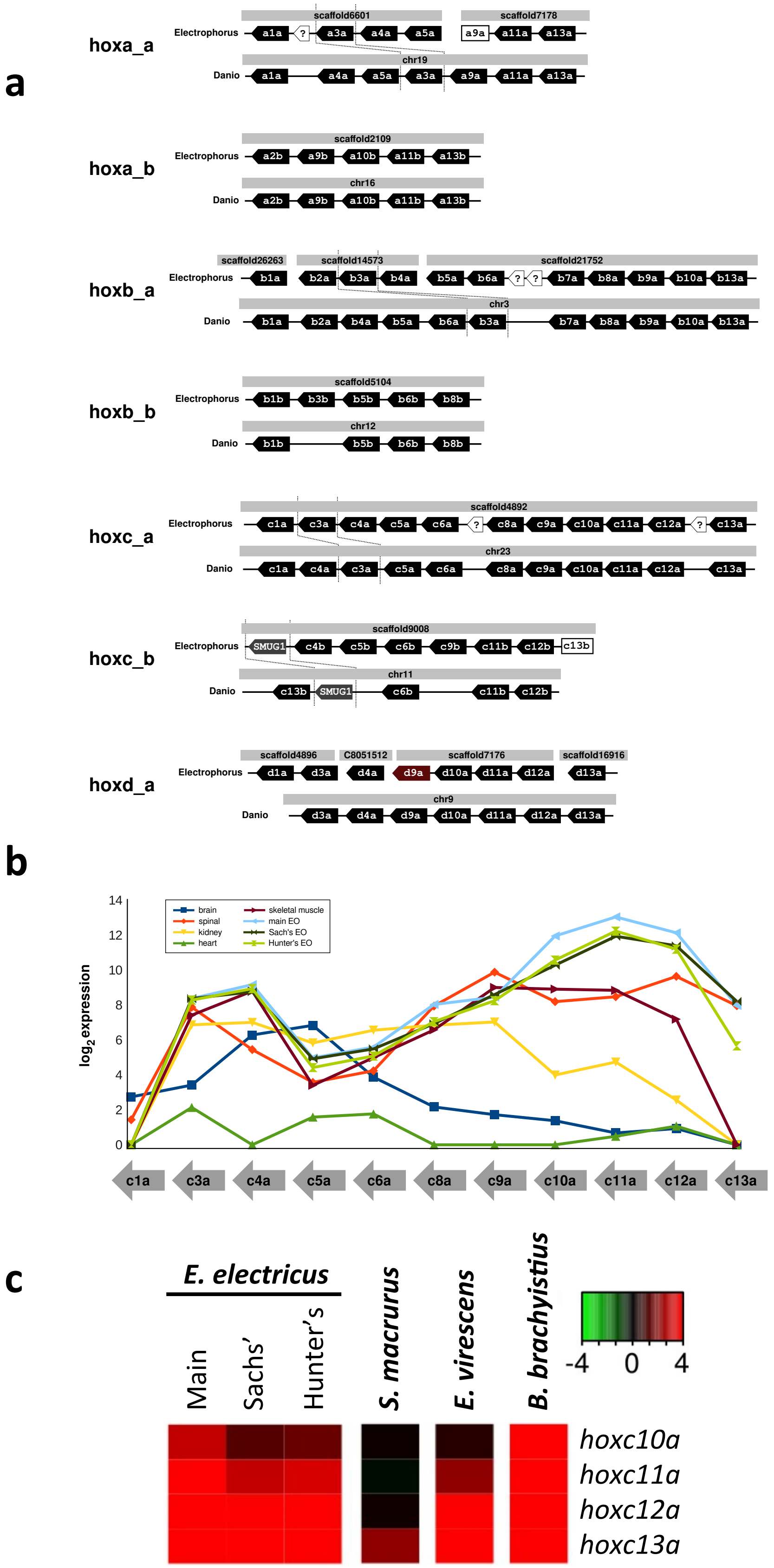
a
hoxa_a
hoxa_b
hoxb_a
hoxb_b
hoxc_a
hoxc_b
hoxd_a
Electrophorus
Danio
scaffold6601
scaffold7178
chr19
scaffold2109
chr16
scaffold26263
scaffold14573
scaffold21752
chr3
scaffold5104
chr12
scaffold4892
chr23
scaffold9008
chr11
scaffold4896
C8051512
scaffold7176
scaffold16916
chr9
SMUG1
b
brain
spinal
kidney
heart
skeletal muscle
main EO
Sach's EO
Hunter's EO
log2 expression
c1a c3a c4a c5a c6a c8a c9a c10a c11a c12a c13a
c
E. electricus
Main
Sachs'
Hunter's
S. macrurus
E. virescens
B. brachyistius
-4 0 4
hoxc10a
hoxc11a
hoxc12a
hoxc13a

Figure S2, A
Cluster 1 (skeletal muscle and heart)

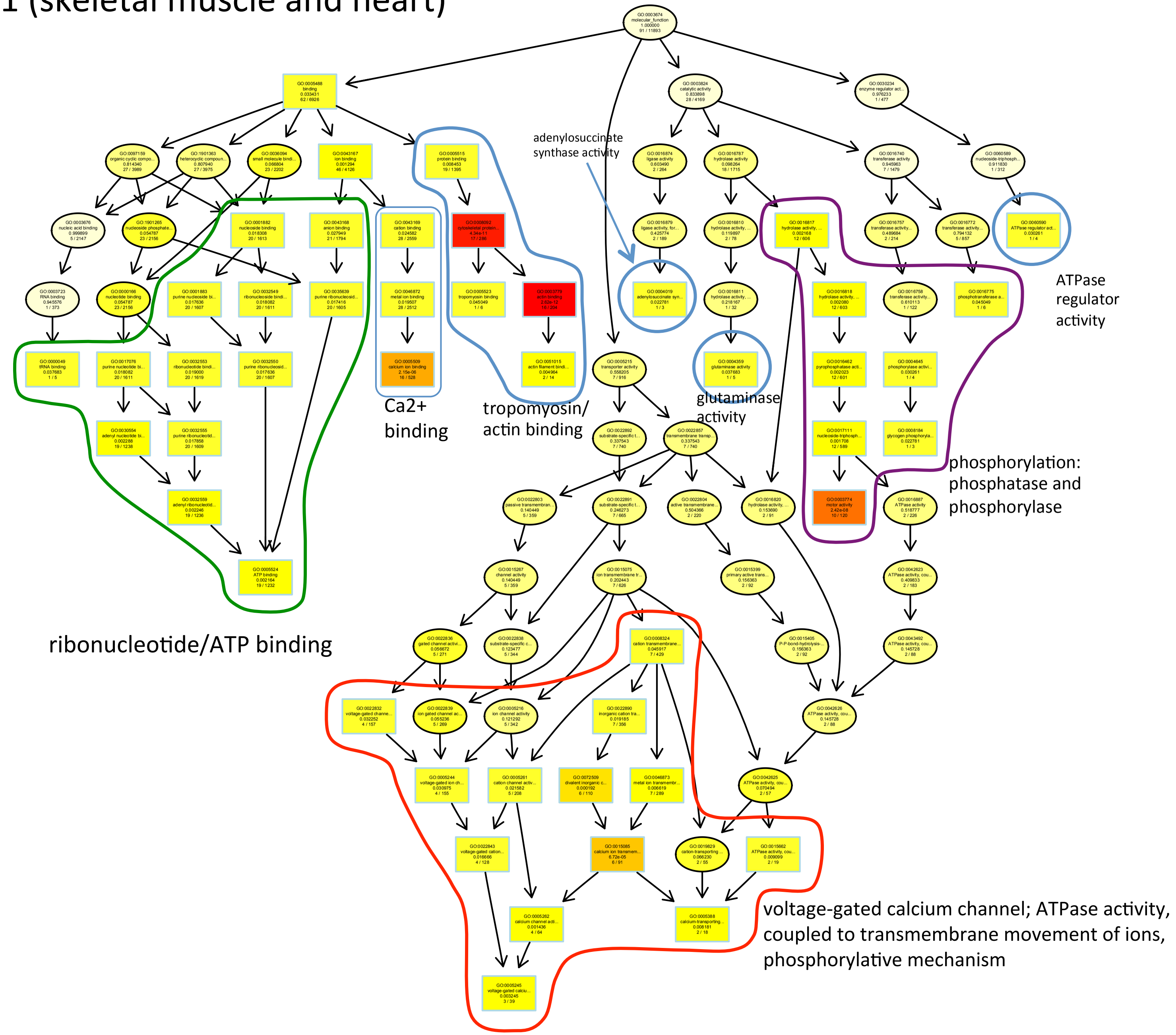

Figure S2, B
Cluster 6 (skeletal muscle and all EOs)

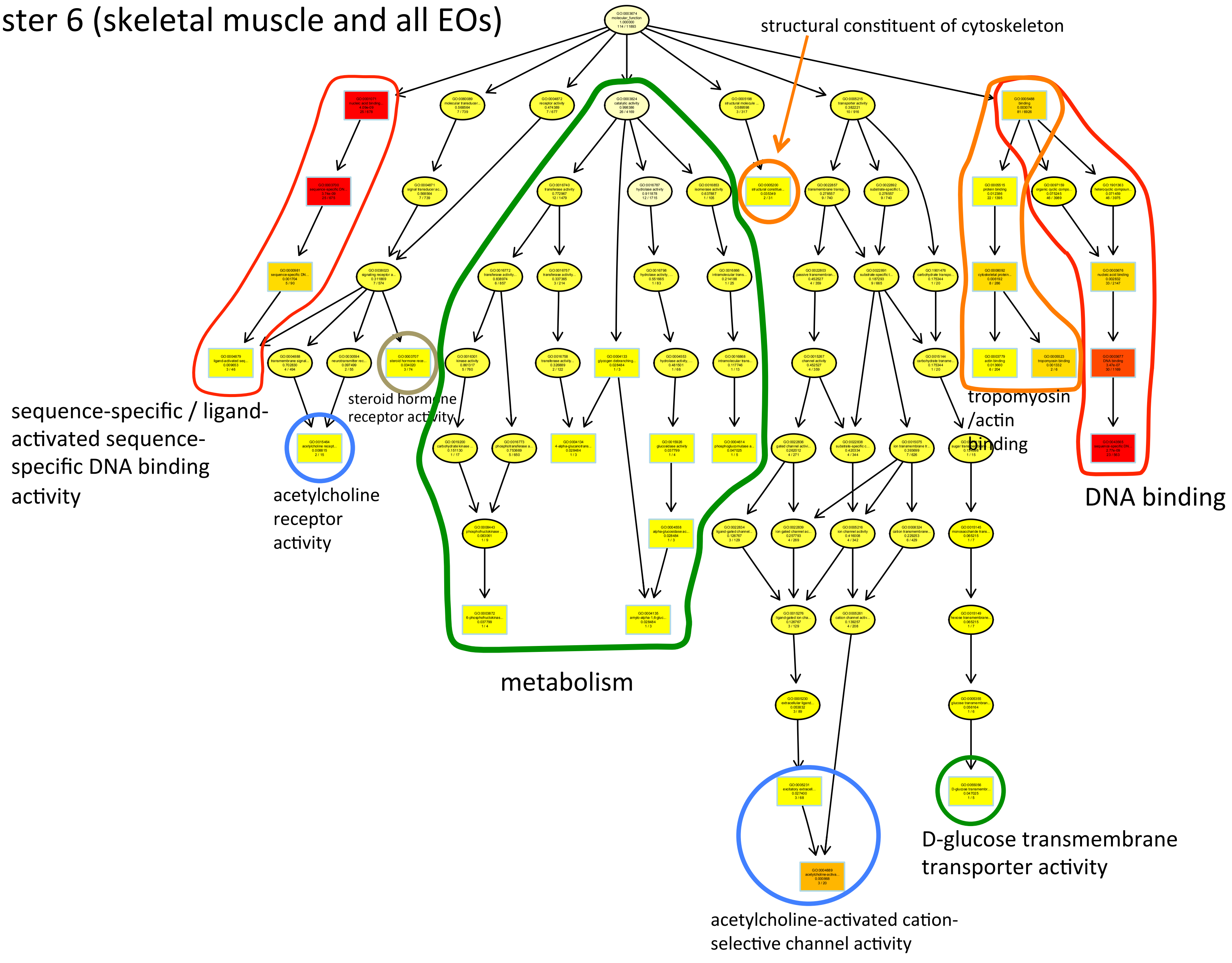

Figure S2, C
Cluster 7 (skeletal muscle, heart, and all EOs)

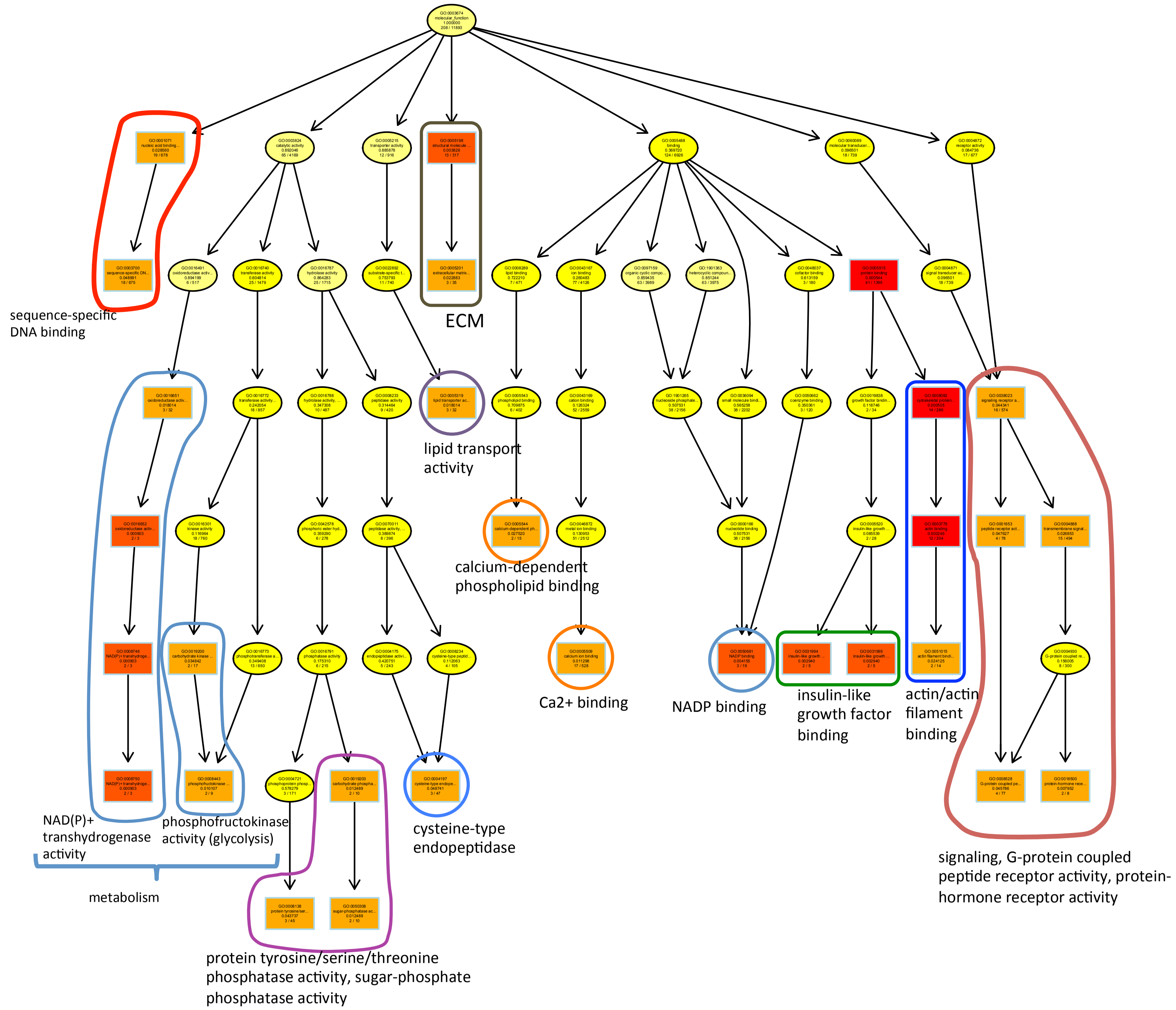

Figure S2, D
Cluster 9 (all EOs)

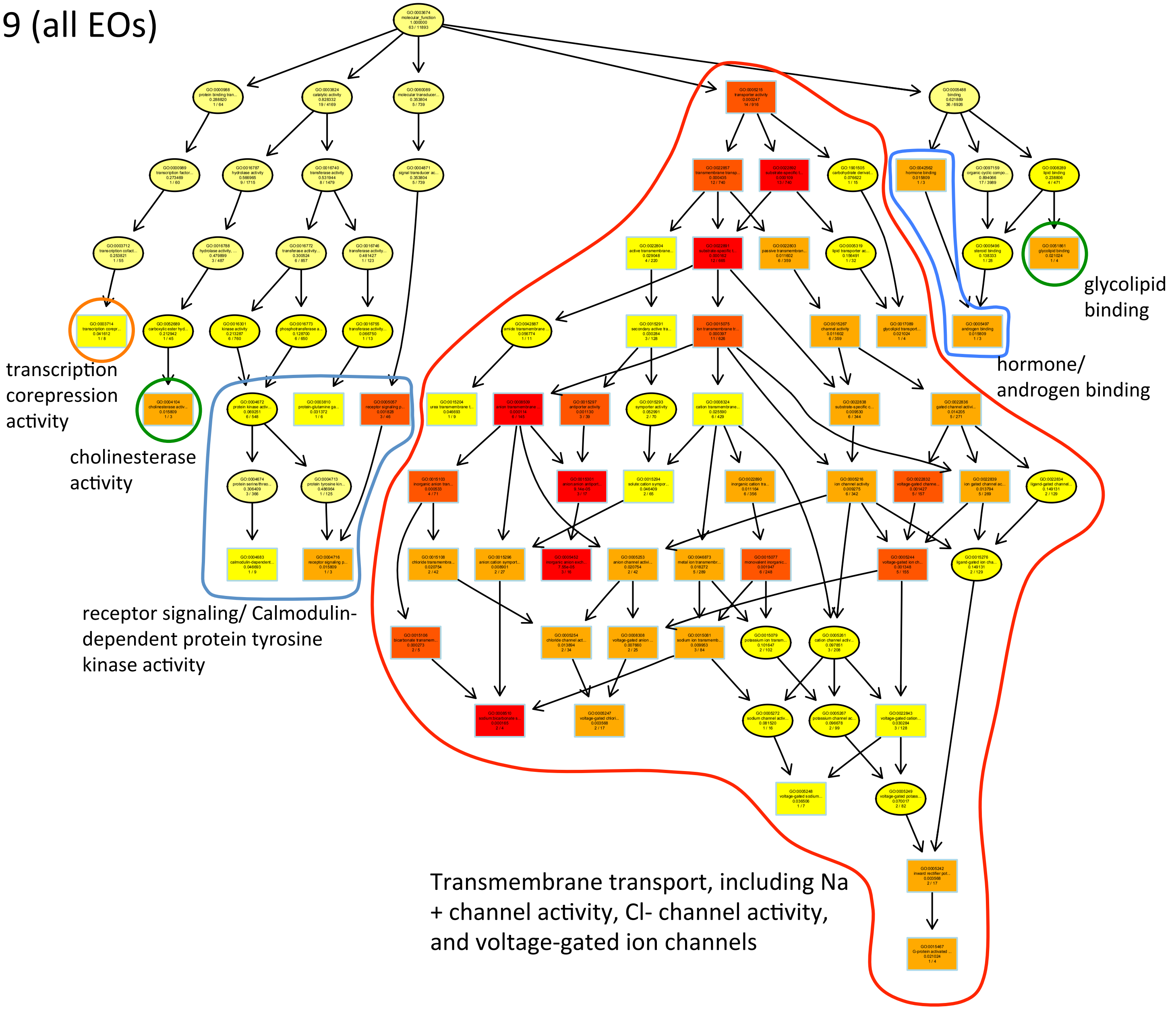

Figure S2, E
Cluster 10 (brain, spinal cord, and all EOs)

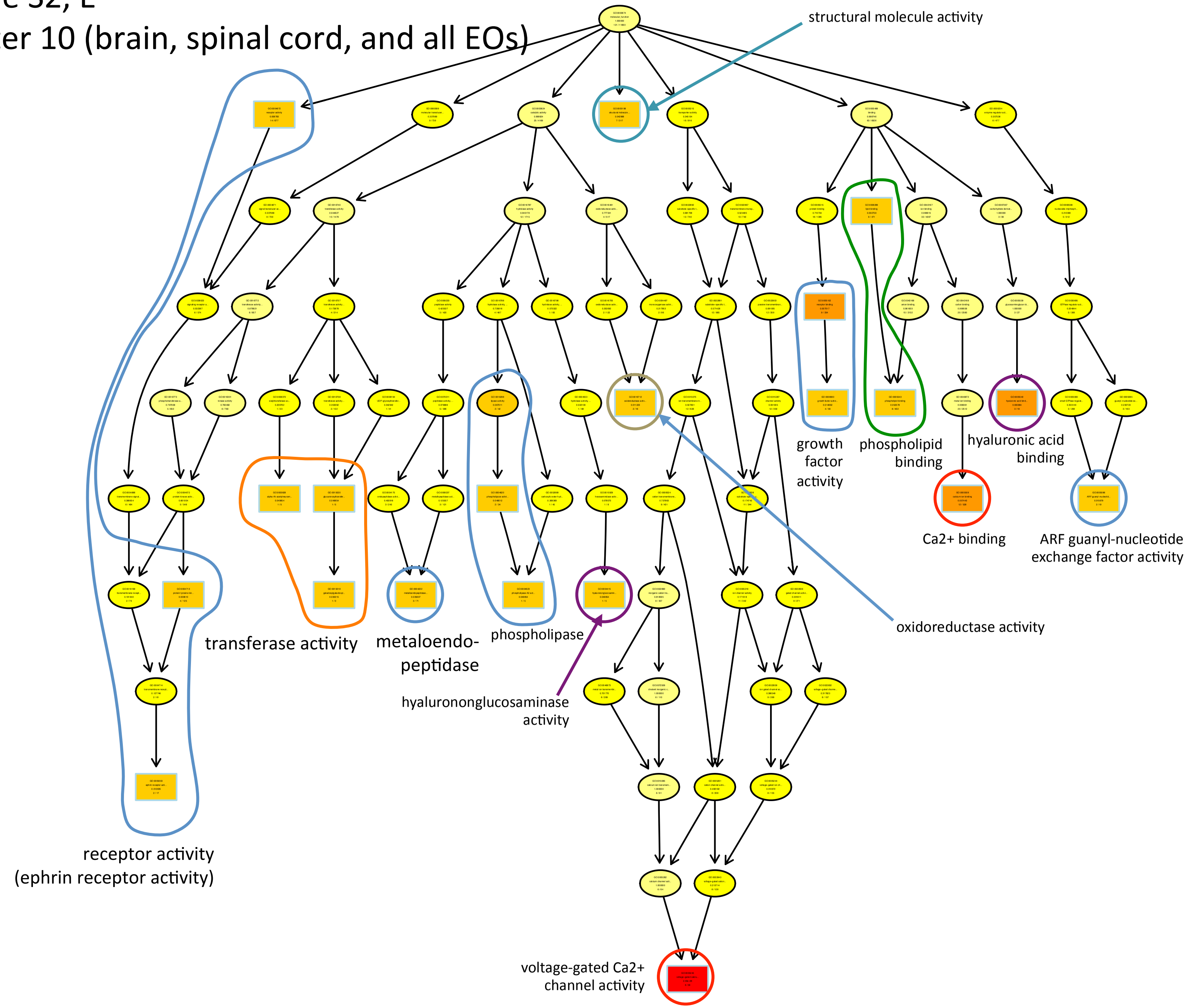

Figure S3

a

**catfish**	AGFFTYFV**I**LAENGFL
**minnow**	AGFFTYFV**I**LAENGFL
**Electrophorus**	AGFFTYFV**V**LAENGFL
**Eigenmannia**	AGFFTYFV**V**LAENGFL
**Sternopygus**	AGFFTYFV**V**LAENGFL
**Malapterurus**	AGFFTYFV**I**LAENGFQ
**Brienomyrus**	AGFFTYFV**I**MAENGFL
**zebrafish**	GGFFTYFV**I**MAENGFL
**tilapia**	AGFFTYFV**I**LAENGFL
**salmon**	AGFFTYFV**I**LAENGFW
**human**	GGFFTYFV**I**LAENGFL
**fly**	AGFFVYFV**I**MAENGFL
**squid (edited)**	AGFFTYFV**V**MAENGWI

b

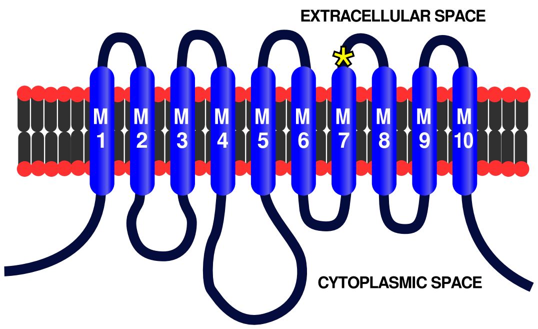

c

	codon	amino acid
squid mRNA	gtc	V
squid gDNA	atc	I

	codon	amino acid
eel mRNA	gtc	V
eel gDNA	gtc	V

Figure S4

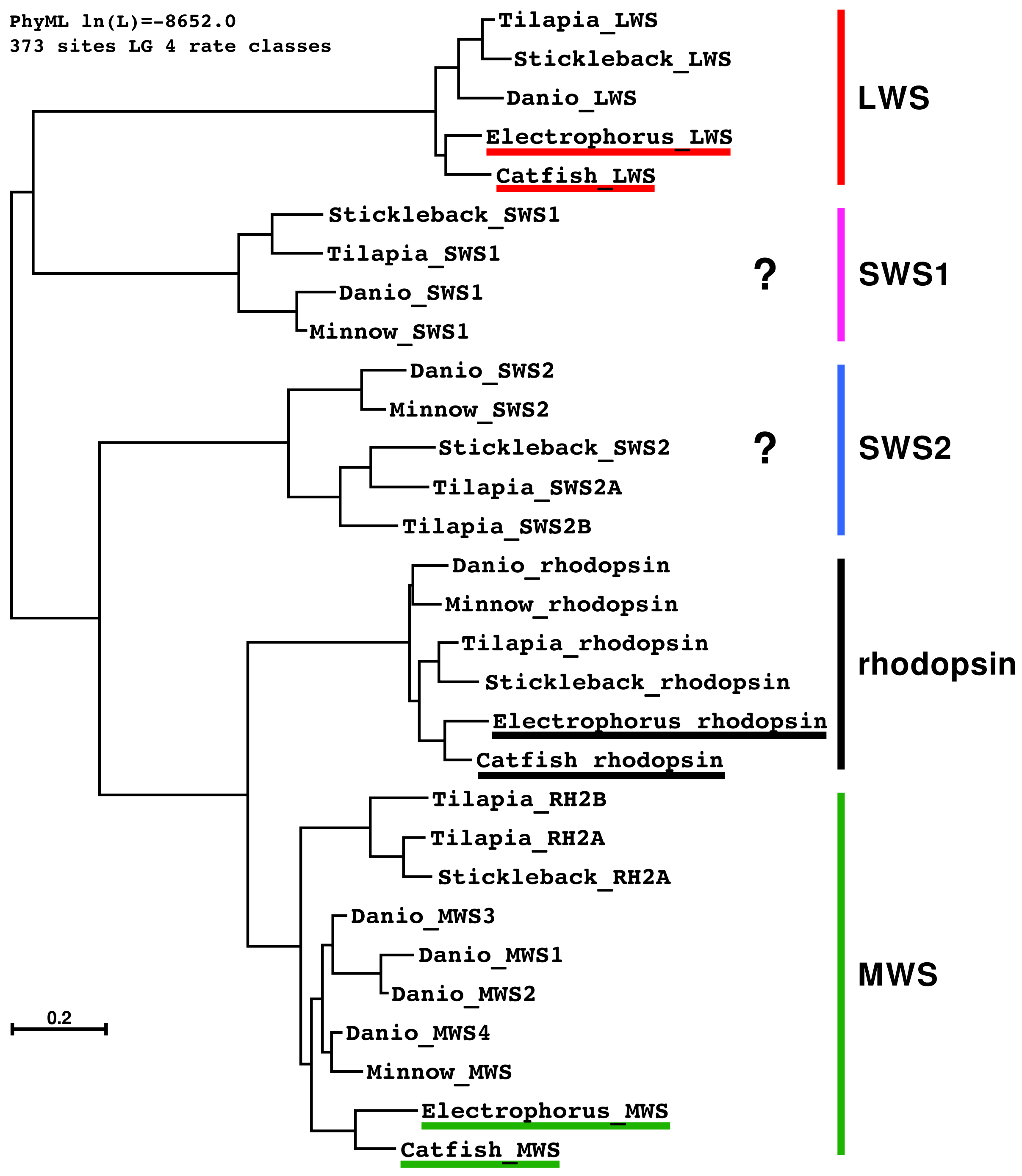
PhyML ln(L)=-8652.0
373 sites LG 4 rate classes
Tilapia_LWS
Stickleback_LWS
Danio_LWS
Electrophorus_LWS
Catfish_LWS
Stickleback_SWS1
Tilapia_SWS1
Danio_SWS1
Minnow_SWS1
Danio_SWS2
Minnow_SWS2
Stickleback_SWS2
Tilapia_SWS2A
Tilapia_SWS2B
Danio_rhodopsin
Minnow_rhodopsin
Tilapia_rhodopsin
Stickleback_rhodopsin
Electrophorus_rhodopsin
Catfish_rhodopsin
Tilapia_RH2B
Tilapia_RH2A
Stickleback_RH2A
Danio_MWS3
Danio_MWS1
Danio_MWS2
Danio_MWS4
Minnow_MWS
Electrophorus_MWS
Catfish_MWS
0.2
?
?
LWS
SWS1
SWS2
rhodopsin
MWS

Table S1

a.

E. electricus mRNA-Seq, Illumina HiSeq 2000	
**Tissue**	**Read count**
Brain	108433089
Spinal	114932446
Heart	116081594
Skeletal muscle	113766333
Main EO	118795559
Sachs' EO	116426272
Hunter's EO	110142469
Kidney (paired reads)	154133686

b.

E. electricus miRNA-Seq, Illumina HiSeq 2000	
**Tissue**	**Read Count**
Brain	13473215
Spinal	19761253
Heart	13332197
Skeletal muscle	15603748
Main EO	14274910
Sachs' EO	17304883
Hunter's EO	11974351
Kidney (paired reads)	101853001

c.

S. macrurus miRNA-Seq, Illumina HiSeq 2000	
**Tissue**	**Read Count**
EO	64713588
Skeletal muscle	73223556

Table S2

	Electrophorus electricus	*Danio rerio*	Medaka (*Oryzias latipes*)	Platyfish (*Xiphophorus maculatus*)	Stickleback (*Gasterosteus aculeatus*)	Fugu (*Takifugu rubries*)	Green Spotted Pufferfish (Tetraodon nigroviridis)	Tilapia (*Oreochromis niloticus*)	*Homo sapiens*	Mouse (*Mus musculus*)
mean exon length	283	244	155	236	163	152	149	229	318	320
mean intron length	1099	2962	1233	1304	781	645	506	1410	7170	5493
mean CDS length	1664	1505	1454	1640	1477	1898	1638	1783	1243	1419
mean transcript length	2992	2072	1551	2583	1655	1917	1702	2534	2106	2424
mean length 5' UTR	274	170	117	302	148	111	110	363	236	205
mean length 3' UTR	1058	616	86	989	185	16	83	818	821	936
number of genes analyzed	23736/19039	26145	19686	20366	20774	18510	19589	21437	22682	22695
